# INCANT: a transnational randomized trial of Multidimensional Family Therapy versus treatment as usual for adolescents with cannabis use disorder

**DOI:** 10.1186/1471-244X-10-28

**Published:** 2010-04-09

**Authors:** Henk Rigter, Isidore Pelc, Peter Tossmann, Olivier Phan, Esther Grichting, Vincent Hendriks, Cindy Rowe

**Affiliations:** 1 Department of Public Health, Erasmus MC, Rotterdam, the Netherlands; 2 Department of Child and Adolescent Psychiatry, LUMC, Leiden, the Netherlands; 3 Department of Psychiatry, CHU Brugmann, Université Libre de Bruxelles, Brussels, Belgium; 4 Delphi-Gesellschaft für Forschung, Berlin, Germany; 5 Centre Emergence, Institut Mutualiste Montsouris; Inserm U669; Université Paris-Sud et Paris Descartes; UMR-S0669; Paris, France; 6 Institut für Sucht- und Gesundheitsforschung, Zürich, Switzerland; 7 PARC, Parnassia Addiction Research Centre, The Hague, the Netherlands; 8 Center for Treatment Research on Adolescent Drug Abuse, University of Miami Miller School of Medicine, USA

## Abstract

**Background:**

In 2003, the governments of Belgium, France, Germany, the Netherlands and Switzerland agreed that there was a need in Europe for a treatment programme for adolescents with cannabis use disorders and other behavioural problems. Based on an exhaustive literature review of evidence-based treatments and an international experts meeting, Multidimensional Family Therapy (MDFT) was selected for a pilot study first, which was successful, and then for a joint, transnational randomized controlled trial named INCANT (INternational CAnnabis Need for Treatment).

**Methods/design:**

INCANT is a randomized controlled trial (RCT) with an open-label, parallel group design. This study compares MDFT with treatment as usual (TAU) at and across sites in Brussels, Berlin, Paris, The Hague and Geneva. Assessments are at baseline and at 3, 6, 9 and 12 months after randomization. A minimum of 450 cases in total is required; sites will recruit 60 cases each in Belgium and Switzerland, and a maximum of 120 each in France, Germany and the Netherlands.

Eligible for INCANT are adolescents from 13 through 18 years of age with a cannabis use disorder (dependence or abuse), with at least one parent willing to take part in the treatment. Randomization is concealed to, and therefore beyond control by, the researcher/site requesting it. Randomization is stratified as to gender, age and level of cannabis consumption.

Assessments focus on substance use; mental function; behavioural problems; and functioning regarding family, school, peers and leisure time.

For outcome analyses, the study will use state of the art latent growth curve modelling techniques, including all randomized participants according to the intention-to-treat principle.

INCANT has been approved by the appropriate ethical boards in Belgium, France, Germany, the Netherlands, Switzerland, and the University of Miami Miller School of Medicine. INCANT is funded by the (federal) Ministries of Health of Belgium, Germany, the Netherlands, Switzerland, and by MILDT: the Mission Interministerielle de Lutte Contra la Drogue et de Toximanie, France.

**Discussion:**

Until recently, cannabis use disorders in adolescents were not viewed in Europe as requiring treatment, and the co-occurrence of such disorders with other mental and behavioural problems was underestimated. This has changed now.

Initially, there was doubt that a RCT would be feasible in treatment sectors and countries with no experience in this type of study. INCANT has proven that such doubts are unjustified. Governments and treatment sites from the five participating countries agreed on a sound study protocol, and the INCANT trial is now underway as planned.

**Trial registration:**

ISRCTN51014277

## Background

In 1999, the (junior) Ministers of Health of five Western European countries - Belgium, France, Germany, the Netherlands, and Switzerland - concluded that their countries were fighting each other over cannabis policies without sufficient scientific evidence to support any view. They agreed to combine scientific efforts. Based on a systematic literature review and the recommendations of an international group of experts [1], the Five-Countries Action Plan for Cannabis Research was adopted in April 2003. It stressed the need of a transnational trial to test an outpatient treatment of cannabis use disorder in youth who may have other problems as well. The Plan acknowledged that adolescents are sensitive to developing cannabis use disorder, which is not easily overcome without treatment [2].

The treatment selected in the Action Plan was Multidimensional Family Therapy (MDFT), developed since 1985 by Liddle and co-workers at the Center for Treatment Research on Adolescent Drug Abuse (CTRADA), University of Miami Miller School of Medicine [2]. MDFT is a family based outpatient treatment programme for adolescent problem behaviour. The term 'multidimensional' reflects the assumption that each major domain in the life of an adolescent may contribute to the incidence and persistence of behavioural problems (through risk factors) and may help in resolving such problems (through protective factors). The life domains include the youth itself, parent, family, friends and peers, school and work, and leisure time. The therapist conducts therapy sessions - with multiple therapeutic alliances: with both the adolescent and the parents -, but also sets out to improve life domain conditions for the adolescent and the family in an outreaching and pragmatic fashion. MDFT views family functioning as instrumental in creating new, developmentally adaptive lifestyle alternatives for the adolescent. Skills training includes substance use relapse prevention, family communication, and parenting. MDFT has been tested with success in different adolescent populations, doses and treatment delivery settings [3,4].

Once it had been decided that a trial was called for, implementation hurdles had to be overcome and confidence in the feasibility of a trial had to be boosted. Although the existence of cannabis use disorder among adolescents had been accepted at the time INCANT was planned, there were quite a few policy makers and therapists who thought that youth with such a disorder, and their families, would not be interested in seeking help, let alone in joining a trial. It was feared that INCANT would fail in recruiting enough subjects, because a (real-world) RCT was still exotic and controversial in Western European youth care at the time. Also, there was concern that a treatment like MDFT, because it is manual-based and time-limited, would stand no chance in countries such as France with a dominant psycho-analytic treatment tradition. Similarly, MDFT was thought to meet opposition in Germany, where treatment of substance abusing adolescents often lasted for more than 1 year. Further, therapists from some of the participating countries - such as Switzerland - believed that mandatory urine tests of alcohol and drug use, which are common practice in American addiction care and a recommended part of MDFT, would not be acceptable to European adolescents.

In view of all this, the five countries carried out a pilot study first to examine the feasibility of a trial of MDFT in Western Europe. Therapists from all participating countries were successfully trained to adequate levels of MDFT adherence and competence as specified in the treatment manual [5]. The potential for recruiting cases for treatment and for study purposes appeared to be promising, and substance urine tests were accepted by virtually all families considered in the pilot study. MDFT could be properly applied, despite variation between the five European countries in mainstream theoretical treatment orientation, personnel requirements, and reimbursement policies.

Because of the generally positive results of this pilot [6], the countries had a protocol prepared for a main study, named INCANT (INternational CAnnabis Need of Treatment). INCANT was to be organized on a transnational basis, with input from all participating countries and with the prospect of helping to create a joint European treatment research infrastructure.

We here report on the design of INCANT.

## Methods/Design

### Design

INCANT is a multicentre phase III(b) randomized controlled trial with an open-label, parallel group design. This study compares MDFT with treatment as usual (TAU) at and across sites in Brussels, Berlin, Paris, The Hague and Geneva. Assessments are at baseline (immediately before randomization) and at 3, 6, 9 and 12 months after randomization.

### Approval

INCANT has been approved by the Ethical Board of Brugmann University Hospital (Belgium), the committee for public law issues of the Chamber of Psychological Psychotherapists and Child and Adolescent Therapists in the state of Berlin (Germany), the Hotel-Dieu Committee for the Protection of Human Subjects in Biomedical Research (CCPPRB; France), the medical-ethical committee for research in mental health care settings (METiGG kamer Noord; the Netherlands), the Ethical Board for Clinical and Outpatient Research (Medical Association of the Geneva Canton; Switzerland), and by the Institutional Review Board of the University of Miami Miller School of Medicine. These boards monitor the progress of the study in terms of recruitment, drop-out and the possible incidence of untoward events.

### Treatment centres

In 2003, government representatives from Belgium, France, Germany, the Netherlands and Switzerland nominated candidate youth outpatient treatment centres for taking part in the pilot study preceding INCANT. The project leader (HR) and CTRADA staff from Miami visited the nominated centres. They selected the department of psychiatry of Brugmann University Hospital in Brussels; Therapieladen in Berlin; Centre Emergence in Paris with suburban CEDAT (Conseils Aide et Action contre le Toximanie) sub-sites in Mantes la Jolie and St Germain en Laye; and the twinning sites of Parnassia Brijder (Mistral, youth addiction care) and De Jutters (Palmhuis, youth forensic care) in The Hague. All these sites did well in the pilot study [6] and have joined the INCANT trial. In Switzerland, the pilot study sites in Zurich, Basle and Bern were replaced by Phénix (Geneva) for the actual trial, as the potential for recruiting substance abusing adolescents was better there.

### Participants

Eligible for INCANT are adolescents of either sex, from 13 through 18 years of age, with a cannabis use disorder (dependence or abuse), with at least one parent willing to take part in the treatment. The word 'parent' denotes any legal representative of the adolescent (including step or foster parent, or guardian). We use the singular 'parent' here, also including the plural 'parents'. Adolescent and parent together are referred to as a 'case'.

Adolescents are ineligible if unable to understand - IQ lower than 70 - the local language, unable to attend outpatient sessions, or if suffering from a mental or behavioural disorder requiring inpatient treatment. Parents (and therefore cases) are ineligible if unable to understand the local language or attend sessions.

Informed consent for study participation is obtained from both adolescent and parent.

### Sample size

We carried out power calculations to determine the number of subjects needed to establish treatment effects on substance use measures within and across sites. To this end, we applied Monte Carlo simulation techniques with latent growth curve models [7]. A large number of samples were drawn, systematically varying effect size estimates, and a model was constructed for each sample. Each simulation tested a linear growth model for continuous outcomes with four time points (0, 6, 9 and 12 months), representing the four major INCANT assessment points. The result of interest was the regression coefficient between a dichotomous covariate representing treatment condition and the latent slope representing change in substance use over time.

In previous trials of MDFT [[4], for review] the size of the comparative treatment effect - the extent in which MDFT outperformed active control treatments - has generally been in the moderate to high range [8] of *d *= 0.6 or above. Conservatively assuming small effects, the models we generated predicted that power would be above 0.9 if the total cross-site sample consisted of 450 or more cases. For individual site analyses, assuming an effect size of 0.7, a sample of 100 per site would be needed to achieve power of 0.82.

We set the recruitment target at 480 cases. The sites in Germany, France and the Netherlands aim to recruit a maximum of 120 cases each. Because of budget limitations, Belgium and Switzerland settle for 60 cases each, with the intention to contribute to the cross-site statistical analyses.

### Recruitment

There are two recruitment annex baseline measurement meetings. The first session, generally with the adolescent and parent together, is carried out by the clinical supervisor of the treatment centre, except in Brussels where the (clinically trained) INCANT researcher serves as the 'front office'. In this session, all eligibility criteria are checked, including the adolescent's cannabis use, but as yet no diagnosis of cannabis use disorder is set. INCANT is explained, and youth and parent are given study information materials and informed consent forms to read before coming back for the second recruitment session generally held a few days later.

In this second meeting, all (remaining) baseline measurements are conducted, separately for youth and parent. If eligible - diagnosis of cannabis use disorder confirmed -, youth and parent are both invited to sign the respective informed consent form.

### Central database

Each site has one or two researchers authorized to access their own site's internet based location - part of the Erasmus MC managed INCANT central database (open source MySCL) -, but not the locations of the other sites. Only the Erasmus MC database manager has full access to all locations and is mandated to change inputted data if so instructed by the project leader (HR) on behalf of the international committee overseeing the design and execution of the trial, viz., the INCANT Study Team (IST).

When inputting data into the database, each case is identified by a code assigned by the database at the time randomization was requested. The INCANT privacy policy ensures that each researcher locally stores person identifying data in such a way that they are blocked from access by others and not become part of the database.

Each questionnaire or interview, per assessment point, has its own file in the database, formatted using PHP Surveyor version 1.0.

### Randomization

Randomization takes place right after having obtained informed consent.

In Belgium, France, Germany and Switzerland, we stratified the study sample using three dichotomous variables (gender; age [13-14 years vs. 15-18 years]; and level of cannabis use in the past 90 days [74 or fewer days of cannabis consumption vs. 75 or more]). In the Netherlands, we added the stratification variable 'ethnicity' (adolescents classified according to national census definitions as being from indigenous or immigrant descent). In total, across sites and sub-sites, there are 72 strata. For each stratum, the database computer generated 50 independent randomisations. For each site except one in France, we have two randomisation arms (MDFT vs. TAU) and we use block randomisation with randomly permuted blocks of 2 or 4 cases. For one site in France, where there are three randomisation arms (MDFT, TAU and TAU-e; see below), we use blocks of 3 or 6 cases.

Randomization is concealed. A researcher enters new cases into the database, through her site's internet location, as soon as informed consent has been obtained, providing data on the stratification variables. Case code and randomization outcome are given automatically and right away, enabling the researcher to inform the family and to schedule appointments with the proper therapist without delay.

### Blinding

Given the nature of the interventions, local researchers cannot be blinded as to the treatment delivered. Central research staff will be unaware of treatment condition when carrying out analyses to assess outcomes.

### Experimental intervention (MDFT)

MDFT is delivered by individual therapists who are part of teams of 3 - 5 CTRADA certified therapists, with one of them additionally serving as team supervisor.

MDFT is carried out according to the MDFT treatment manual http://kap.samhsa.gov/products/manuals/cyt. MDFT lasts 5 to 7 months, depending on the severity of the case. On average, sessions are scheduled twice a week - in roughly equal proportion to be held with the adolescent, parent and family (adolescent + parent) respectively, and additionally with representatives of other systems (school, work, friends, agencies) present. Sessions can take place at the office, but also at the family's home or any other convenient location. Scheduling sessions is not limited to regular office hours. Each team meets once a week to discuss cases and issues.

### Control condition

INCANT compares MDFT with treatment as usual (TAU). TAU is carried out by the same treatment centres offering MDFT, but procedurally separated to avoid 'contamination' of therapists and participants between the experimental and control conditions.

TAU varies between the participating countries, but has in common motivational interviewing and elements of cognitive-behavioural therapy (CBT) in addition to more general individually-based substance abuse counselling. TAU in Belgium, Germany and Switzerland is characterized by a mixture of CBT and individual drug counselling, and in the Netherlands by a cognitive-behavioural approach tailored to adolescents (Leefstijltraining). The Dutch TAU therapists have received formal training in TAU for the purpose of the trial. In France, MDFT is compared with TAU (a mix of CBT and individual counselling methods as used in daily practice) and TAU-e (TAU-explicit = idem, but then manualized). The French TAU-e therapists have been trained in using the TAU-e manual; the TAU therapists have not received special training for INCANT. Below, French TAU and TAU-e are jointly referred to as 'TAU'.

Across sites, we set minimal requirements for TAU. The control treatment matches MDFT in total duration of the therapy. MDFT and TAU do not differ in assessments and in general procedures such as therapists working in a team; applying substance use urine tests; communication with referral sources and authorities; and occasional referral to additional treatments to deal with psychiatric co-morbidity and medication. Furthermore, MDFT and TAU do not differ in session duration; drug education; and the way the adolescent is individually trained in substance use relapse prevention, with emphasis put on coping with stress, managing anger, increasing assertiveness in interpersonal contacts, and addressing (negative) thoughts about substance use.

TAU sessions are individual (with the adolescent). Parents may be seen alone or in groups, purely for reasons of drug education and mutual support, but any element of systems therapy involving the parent and other systems into the treatment is excluded.

### Monitoring of treatment integrity

In all INCANT conditions, the therapists are required to submit *treatment contact logs *or data from the centre's treatment register providing the same insight: number, duration, spacing and composition of sessions (therapist meeting whom). These data are used throughout the study to ensure that all therapists meet the pre-set minimum level of treatment intensity. In MDFT, these logs are monitored by CTRADA (University of Miami) staff to ensure fidelity to MDFT parameters of contact with adolescent, parent, family, and external systems sessions.

Based on treatment adherence evaluation guidelines [5], 25% of MDFT cases are selected for transcribing one family session, which is translated in English to allow CTRADA to rate these sessions for MDFT treatment adherence with a validated 7-point Likert scale targeting 16 therapists' interventions crucial to MDFT [9].

### Therapists

All MDFT (n = 25) and TAU therapists (n = 29) are experienced in treating adolescents with multiple problem behaviour. MDFT therapists are similar to TAU therapists with regard to age (MDFT vs. TAU across sites: 42 vs. 40 years of age) and gender (48% male in MDFT, and 35% in TAU).

CTRADA trained the sites' MDFT teams in 2004 - 2005, with plenary training weeks in Europe and Miami; evaluation of MDFT family sessions and session logs; bimonthly telephone consultation calls; and site visits by CTRADA staff. Booster training was given in 2006 and 2007. TAU training in the Netherlands and TAU-e training in France were delivered locally by senior clinical staff from the treatment centre (France) or by a CBT training unit (the Netherlands).

### Remuneration

The adolescents but not the parents are remunerated in local currency, either by voucher or in cash, for completing follow-up assessments, for a total of € 60 - 70 accumulated across all follow-up assessments. No remuneration is given in Belgium and France, where this would run counter to legal requirements.

### Assessments

Assessments take place at baseline and at 3, 6, 9 and 12 months post-randomization, at the treatment centre, the home of the family or any other convenient place. Additional information is gathered by phone or mail. Questionnaires are self-administered by the adolescent or parent, or if required completed by a researcher, who has been trained by INCANT project staff and is working under the guidance of three Instruction Manuals (for baseline, 3-months FU, and later FU, respectively; http://incant.eu). Table 1 shows the questionnaires and interviews delivered.

**Table 1:** Measures used in INCANT at baseline and at four post-randomization follow-up points

Surveys	Baseline	Month 3	Month 6	Month 9	Month 12
	**Adolescent**				

Cannabis section, ADI-Light	•				•
Alcohol section, ADI-Light	•				•
Other drugs, ADI-Light	•				•
Urine analyses of substance use	•	•	•	•	•
TLFB	•	•	•	•	•
Adolescent Interview	•		•		•
Life Events	•				•
PEI	•		•	•	•
Cannabis section, ADI-Light	•				•
Alcohol section, ADI-Light	•				•
Other drugs, ADI-Light	•				•
FES	•		•	•	•
YSR	•		•		•
Treatment satisfaction, adolescent			•		

	**Parent**				

Parent Interview	•		•		•
CBCL	•		•		•
Treatment satisfaction, parent			•		

Abbreviations: ADI = Adolescent Diagnostic Interview, CBCL = Child Behavior Check List, FES = Family Environment Scale, PEI = Personal Experience Inventory, YSR = Youth Self-Report.

### Study hypotheses

INCANT addresses a number of research questions:

#### Primary outcomes

Does MDFT exceed TAU in reducing the use of cannabis and the prevalence of (symptoms of) cannabis disorders? We assume that adolescents assigned to MDFT will decrease their use of cannabis more than adolescents in TAU between baseline and 6-months follow-up assessments. This treatment gain is expected to be maintained better in MDFT than in TAU in the period between 6- and 12-months follow-up.

Moreover, youth assigned to MDFT will be less likely to meet diagnostic criteria of cannabis disorders between baseline and the 12-months follow-up assessment than TAU youth.

#### Secondary outcomes

An important secondary outcome is the extent in which the treatments succeed in engaging and retaining cases into the respective intervention programme. We assume that MDFT will do better than TAU in this respect.

Further, we hypothesize that MDFT exceeds TAU in attenuating established risk factors for persistence of cannabis disorders: other substance use, internalizing and externalizing mental disorder symptoms, family dysfunction, school problems, delinquency. For each of the factors mentioned, a hypothesis has been formulated and measures have been selected.

Finally, we examine the degree in which MDFT and TAU are appreciated by adolescents and parents, assuming that MDFT will receive higher satisfaction ratings than TAU.

### Measures

Table 1 gives an overview of the instruments - questionnaires and structured interviews - to be applied at baseline and four follow-up assessment points, distinguishing measures administered to youth and parent.

#### Background and demographic information

The *Parent and Adolescent Interviews *[9] have been tailored to gather demographic data on gender, age and ethnicity, and on family composition, history of familial drug use and mental health problems, adolescent substance use history and court involvement, treatment history and service utilization, school functioning, peer relationships, and pastime activities.

#### Primary outcomes: cannabis use

Cannabis use and other substance use disorders are assessed with the *Adolescent Diagnostic Interview-Light *(ADI-Light; [10]). This brief structured, multi-axial interview is based on DSM-IV criteria for substance use disorders in adolescents. At baseline, an ADI-Light established diagnosis of recent cannabis use disorder was required to enrol the case into INCANT.

We measure the frequency of adolescents' cannabis use with the *Timeline Follow-Back *method (TLFB; [11] as adapted and validated for adolescents [12]. The TLFB obtains retrospective reports of daily cannabis use for the 90-day period prior to each assessment, using a calendar and other memory prompts to stimulate recall.

Adolescents' preoccupation with and motivation for substance use are recorded with the Personal Involvement with Chemicals Scale from the *Personal Experiences Inventory *(PEI). The psychometric properties of this scale are excellent. A reliability score of α = 0.97 has been reported [13].

#### Secondary outcomes: adolescents' psychosocial functioning

We assess adolescents' internalizing, externalizing, and psychotic symptoms with the *Youth Self Report *(YSR). This instrument is reliable and valid across a variety of studies, populations, and languages (including Dutch, German and French) [14,15]. We also apply the 'parent version' of the YSR, which is called CBCL (*Child Behavior Checklist*). Like the YSR, the CBCL has excellent (test-retest, internal consistency, inter-rater) reliability and (construct, concurrent, discriminant) validity in various languages [e.g., [14,16]].

#### Secondary outcomes: family functioning

Family conflict and cohesion are measured with the respective sub-scales from the *Family Environment Scale *(FES), a widely used and well validated self-report measure [17], completed by the teen. The FES has adequate psychometric properties. The Conflict and Cohesion parts have good reliability (α = 0.75 and α = 0.78, respectively) [17,18].

#### Secondary outcomes: treatment satisfaction

At 6-months follow-up, corresponding to the end of a full course of treatment, adolescents and parents each complete the *Satisfaction Scale*, which measures satisfaction with treatment received along four dimensions: (1) access to and convenience of treatment arrangements, (2) adolescent's treatment process and relationship with the therapist, (3) parent and family services, and (4) global satisfaction. These scales have adequate reliability and validity [19].

### Analyses

The analyses will be based on the intent-to-treat principle, such that all cases will be assessed at all time points regardless of therapy and therapy dosage received.

We will pool the data from all subjects from all sites for analyses with country/site as a covariate. If differences between sites are statistically significant, within-site analyses will be done for the topics concerned.

Most measures in the trial are repeated (Table 1). For repeated measures we will apply both a mixed model for repeated measurements and Latent Growth Curve Modelling (LGM). LGM serves to model individual differences in change as measured by instruments such as the TLFB and PEI. LGM has the advantage of charting individual change trajectories [7] while producing unbiased estimates when data are missing [20]. Change trajectories will be evaluated across all assessment points.

The LGM models are statistically equivalent to random coefficient regression models.

As for MDFT treatment adherence, we will use equivalence testing procedures to compare the mean MDFT adherence scores to benchmark values for therapists trained in U.S.-based trials of MDFT, to see if they are statistically equivalent. We will use an a priori equivalence interval of +/- 20 percent. A 90% confidence interval will be calculated around the mean difference between the adherence scores in INCANT and the benchmark values. If the values for the 90% confidence interval fall within the a priori equivalence interval, the scores are considered to be statistically equivalent [21].

To meet the challenges of data analyses, a statistical workgroup has been formed consisting of specialists from Erasmus MC, CTRADA, INSERM (France) and Delphi (Germany). The workgroup will report to the INCANT Study Team.

## Discussion

There are differences in mainstream treatment philosophy between the countries taking part in INCANT. Thanks to a pilot study and intensive international consultation and training, these differences eventually appeared to be surmountable or at least not strong enough to block the trial here reported. INCANT is firmly underway, with steady enrolment of cases.

INCANT is more than a treatment trial. In a way, it is also a social and cultural experiment. At first, European officials thought that MDFT, as it originates from the USA, might be at odds with European practices and traditions. However, while preparing for INCANT the differences between European countries appeared to be bigger than the difference between any of them and Miami. Nevertheless, MDFT proved to be adaptable to European treatment settings in all of the five countries, such that procedures are mutually comparable (except for referral source).

In some of our five countries, there was hardly any experience, if at all, with conducting an RCT in youth (addiction, mental, forensic) care. Making referral authorities, treatment and funding agencies accept the principle of randomization was seen as (almost) a bridge too far. If INCANT yields any comparative treatment effects remains to be seen, but INCANT did succeed in making a trial happen in rather uncharted territory.

## Abbreviations

ADI: Adolescent Diagnostic Interview; CBCL: Child Behavior Checklist; CBT: cognitive-behavioural therapy; CTRADA: Center for Treatment Research on Adolescent Drug Abuse; FES: Family Environment Scale; INCANT: International Cannabis Need of Treatment study; IST: INCANT Study Team; MDFT: Multidimensional Family Treatment; PEI: Personal Experiences Inventory; TAU: Treatment As Usual; TAU-e: manualized TAU in France (e = 'explicit'); TLFB: Timeline Follow-Back; YSR: Youth Self Report.

## Competing interests

CR trains teams of therapists in MDFT as a consultant. All other authors declare that they have no competing interests.

## Authors' contributions

All authors were substantially involved in the conception of the study. HR and CR designed and coordinated the overall study, whereas IP, PT, OP, VH and EG did set up the study at the respective national sites. All authors are instrumental in collecting and interpreting the data. HR drafted the manuscript with the assistance of CR. The other authors critically revised the text. All have approved the present publication.

## Pre-publication history

The pre-publication history for this paper can be accessed here:

http://www.biomedcentral.com/1471-244X/10/28/prepub
